# Breast cancer combined prognostic model based on lactate metabolism genes

**DOI:** 10.1097/MD.0000000000032485

**Published:** 2022-12-23

**Authors:** Na Lu, Xiao Guan, Wei Bao, Zongyao Fan, Jianping Zhang

**Affiliations:** a Department of General Surgery, The Second Affiliated Hospital of Nanjing Medical University, Nanjing, Jiangsu Province, China; b Department of Urology, The Second Affiliated Hospital of Nanjing Medical University, Nanjing, China.

**Keywords:** bioinformatics, breast cancer, immune infiltration, lactate metabolism genes, prognosis

## Abstract

To investigate the impact of lactate metabolism genes, lactate metabolism-related genes (LMRG), and immune infiltrating cells on the prognosis of breast cancer. LMRG was identified via single-cell sequencing. Immune cell infiltration was obtained by the CIBERSORT method. The prognostic genes were chosen by cox regression and the least absolute selection operator approach. lactate metabolism-associated immune-infiltrating cells was determined by difference analysis. The GSE20685 dataset was used as an external validation cohort. The model’s prognostic usefulness was evaluated utilizing survival, immunological microenvironment, and drug sensitivity assessments. NDUFAF6 was most associated with breast cancer prognosis. We obtained a total of 450 LMRG. SUSD3, IL18, MAL2, and CDKN1C comprised the Model2. NK cell activation was most relevant to lactate metabolism. The combined prognostic model outperformed the individual model, with the area under the curve ranging from 0.7 to 0.8 in all three cohorts. The lactate metabolism-related combination model assisted in evaluating breast cancer prognosis, providing new insights for treatment, particularly immunotherapy.

## 1. Introduction

Breast cancer has become the most frequent malignancy globally and the major cause of cancer mortality among women, placing a heavy burden on global health.^[1]^ With the development of multidisciplinary treatment of breast cancer, comprehensive treatment based on surgery has become the main treatment strategy.^[2]^ However, the overall prognosis remains dismal, especially in patients with advanced stage.^[3]^ Currently available treatments are considered incurable for advanced breast cancer with distant organ metastases.^[4]^ Breast cancer is a disease with many complicated and varied origins that is very heterogeneous.^[5]^ Despite the development of several prognostic techniques, none of them were capable of precisely predicting each patient’s prognosis.^[6]^ Therefore, exploring new prognostic models and therapeutic targets may provide new insights into the treatment.

A cutting-edge sequencing technique called single-cell sequencing offers pertinent data for the characterization of specific tumor cells or immune cells.^[7]^ Single-cell sequencing also highlights intra-tumor heterogeneity and different subpopulations, allowing enumeration and quantification of immune infiltration in tumor tissue.^[8]^ We can comprehend the cellular differentiation and immunological pathways of breast cancer by cell clustering and annotation.^[9]^ Single-cell sequencing data is crucial for examining the properties of each cell subpopulation and the connections between individual cells.^[10]^

Metabolic reprogramming is an important feature of tumors, and aerobic glycolysis is 1 of the important hallmarks of tumor metabolic reprogramming.^[11]^ Glycolysis is the main energy source for tumor cells, which produces large amounts of lactate.^[12–14]^ Lactic acid is a key regulator in malignant tumor signaling pathways, generating an environment that is immunosuppressive and encourages the growth and immune evasion of cancer cells.^[15–17]^ High lactate levels in tumors are associated with tumor metastasis and recurrence.^[18]^ In addition, lactate contributes to tumor inflammatory processes and promotes tumor angiogenesis.^[19]^ Inhibition of lactate metabolism is a potential cancer treatment.^[20]^ However, it is uncertain whether the genes involved in lactate metabolism with related to breast cancer prognosis.

Herein, we 1^st^ selected the lactate metabolism genes associated with breast cancer prognosis. Based on these genes, we identified lactate metabolism-related genes (LMRG) by single-cell sequencing analysis and identified lactate metabolism-associated immune-infiltrating cells (LMIC). Based on the findings of the aforementioned investigation, we created a composite prognosis prediction model for patients with breast cancer. The research helps to guide breast cancer treatment plans.

## 2. Methods

### 2.1. Data collection

From the cancer genome atlas (TCGA) database, clinical and transcriptomic data were downloaded. The workflow type Counts were used. From the gene expression omnibus (GEO) database, we retrieved the breast cancer single-cell sequencing dataset GSE168410. Data from 5 patients were selected. Additionally, we obtained the GSE20685 microarray gene expression profiling dataset from the GEO database. All data were log2 transformed. From the MSigDB database, we collected 288 lactate metabolism genes.

### 2.2. TCGA data process

Getting the count file, the downloaded data were 1^st^ merged and preprocessed using the Perl programming language. With Perl, the gene symbols were altered. The transcriptomic data from TCGA were then matched to lactate metabolism genes. Patients without follow-up days and with insufficient clinical data were excluded. The survival data were matched to lactate metabolism genes.

The TCGA cohort was randomly divided into a training cohort and a test cohort at a 7:3 ratio. We first performed a differential analysis (*P* < .05) to screen for genes that differed between the normal and tumor groups in the training cohort. The univariate COX analysis was then done, and genes were chosen (*P* < .05). These selected lactate metabolism genes were correlated with breast cancer prognosis and used in subsequent analyses. We retained the most significantly different gene and used its expression as Model1.

### 2.3. Data processing of the GSE168410

We screened data from 5 patients from the dataset for single-cell sequencing analysis. We retained cells with between 500 to 4000 genes, fewer than 1% of the ribosomal and erythrocyte genes, and fewer than 5% of the mitochondrial genes. We retained genes that were expressed in at least three cells. Based on the degree of fluctuation in all samples, 6000 genes with the highest fluctuations were identified. We normalized and integrated the samples by the “LogNormalize” method. Then, we performed principal component analysis to reduce dimensionality and used the TSNE method for cluster analysis. The cell types’ annotations were done using the “SingleR” package. The “singler” approach was used to define the cell subsets. Then we imported the lactate metabolism genes associated with breast cancer prognosis. Using the “PercentageFeatureSet” tool, we determined the percentage of these genes in each cell. Based on these genes’ median ratio, the low lactate or high lactate cells were classified. Differential genes between these 2 types of cells were identified by the “FindMarkers” function (*P* < .05). LMRG were assigned to these genes.

### 2.4. Prognostic model based on LMRG (Model2)

First, data on LMRG expression and survival were matched. The univariate COX analysis (*P* < .05) was performed after that. The least absolute selection operator regression approach was used to further choose the LMRG that had prognostic relevance. Then, each patient’s risk score was calculated, and the prognostic model was developed. Using the median, we classified them into high- and low-risk groups. Next, we compared the 2 groups prognoses and examined the accuracy of Model2.

### 2.5. Validation and evaluation of the Model2

The GSE20685 dataset was used as the validation cohort. The Model2 formula was used to compute the risk score for each sample. Afterward, we performed the survival analysis to see if there was a difference in prognosis between the training and test cohort. Meanwhile, to evaluate the efficiency of stratifying samples, we plotted the sample distribution between the 2 groups. Heatmaps were used to compare the Model2 genes’ expression levels. The predictive ability of Model2 was verified using the ROC curve. We performed survival analysis and drew ROC curves to verify the validity of Model2 in the validation cohort.

### 2.6. Functional analysis

Using the “clusterProfiler” package, the gene ontology and kyoto encyclopedia of genes and genomes pathway analyses were carried out. Additionally, we utilized the “gene set variation analysis (GSVA)” package to carry out GSVA. The conclusions were displayed by bar charts (*P* < .05).

### 2.7. Immunoassay and m6A analysis

Model2 and the degree of tumor infiltration were compared using immune infiltration heatmaps and correlation maps. We mainly refer to CIBERSORT and XCELL methods.^[21,22]^ We found a list of m6a-related^[23–25]^ and immune checkpoint-related genes.^[26–29]^ The boxplots represented the analyses’ findings.

### 2.8. Identification of LMIC

Through CIBERSORT analysis, we got the makeup of 22 breast cancer immune cells.^[21]^ The distribution of various immune cell types in breast cancer in each sample was represented by a rainbow graph. Using the median gene expression levels of Model1, patients were split into groups with high and low expression. Differences in immune cell infiltration between the 2 groups were shown using boxplots. We defined these cells as LMIC (*P* < .05). We used its value as Model3.

### 2.9. Combined prognostic model

Based on Model1, Model2, and Model3, the combined prognostic model was established. The correlation coefficient of the combined model was calculated by multivariate COX regression. ROC curves and survival curves were used to examine the predictive performance of the combined model.

### 2.10. Drug sensitivity analysis

The Cancer Genome Project provided us with expression matrices and medication processing data. Using the “pRROpheticPredict” function, the medicines linked to the combined model were obtained (*P* < .00001).^[30]^

## 3. Results

**Figure 1.**
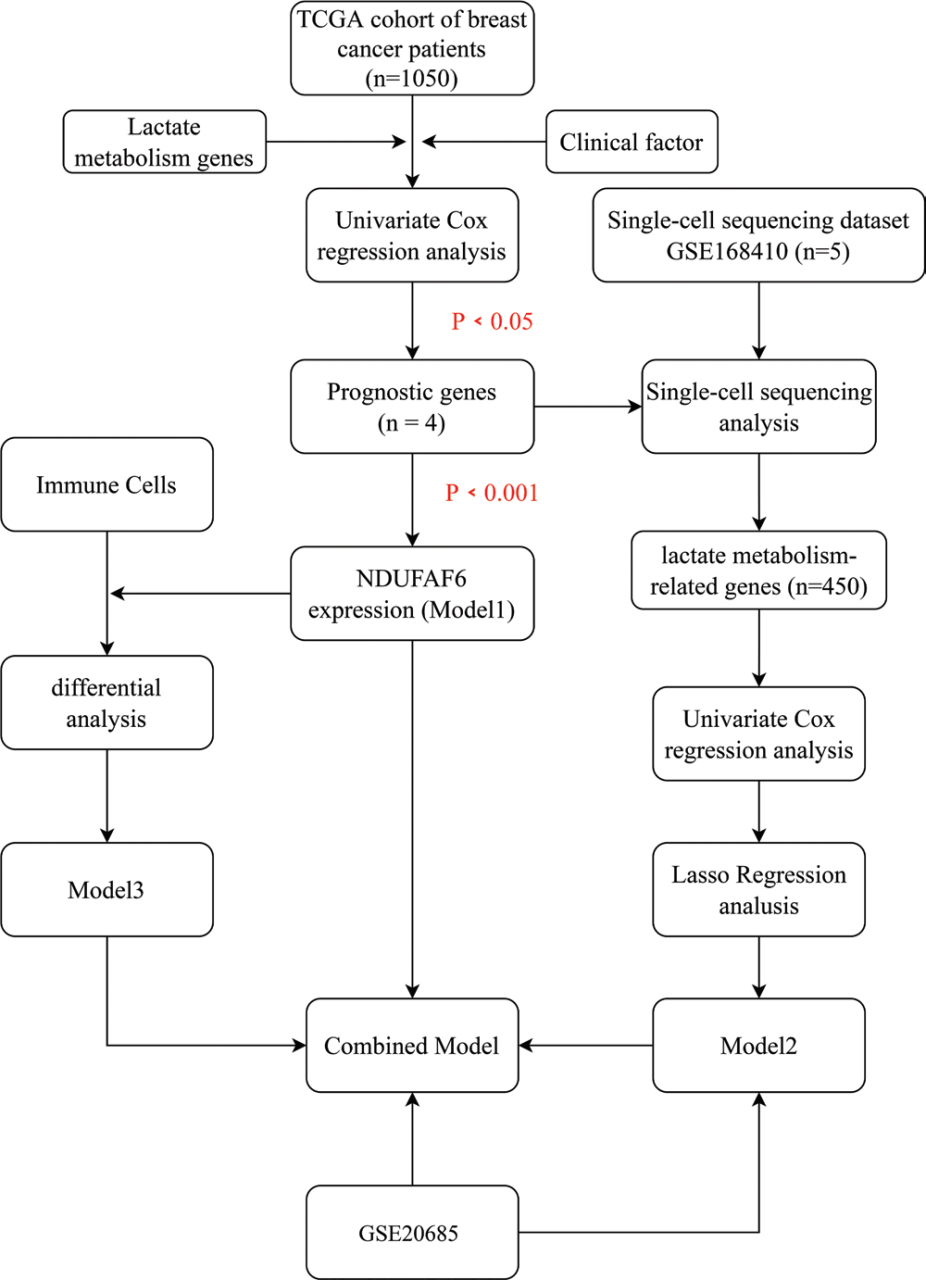
The flowchart of this study.

Figure 1 showed the workflow of this study

### 3.1. Construction of the Model1

We performed differential analysis on the matched data and screened out 31 genes with differences between the normal and tumor groups. We then performed independent prognostic analysis, retaining a total of 4 genes, including TYMP (*P* = .031), PNKD (*P* = .030), SCO2 (*P* = .039), and NDUFAF6 (*P* = .005). We used the NDUFAF6 expression as Model1.

### 3.2. Analysis of the GSE168410 Dataset

**Figure 2.**
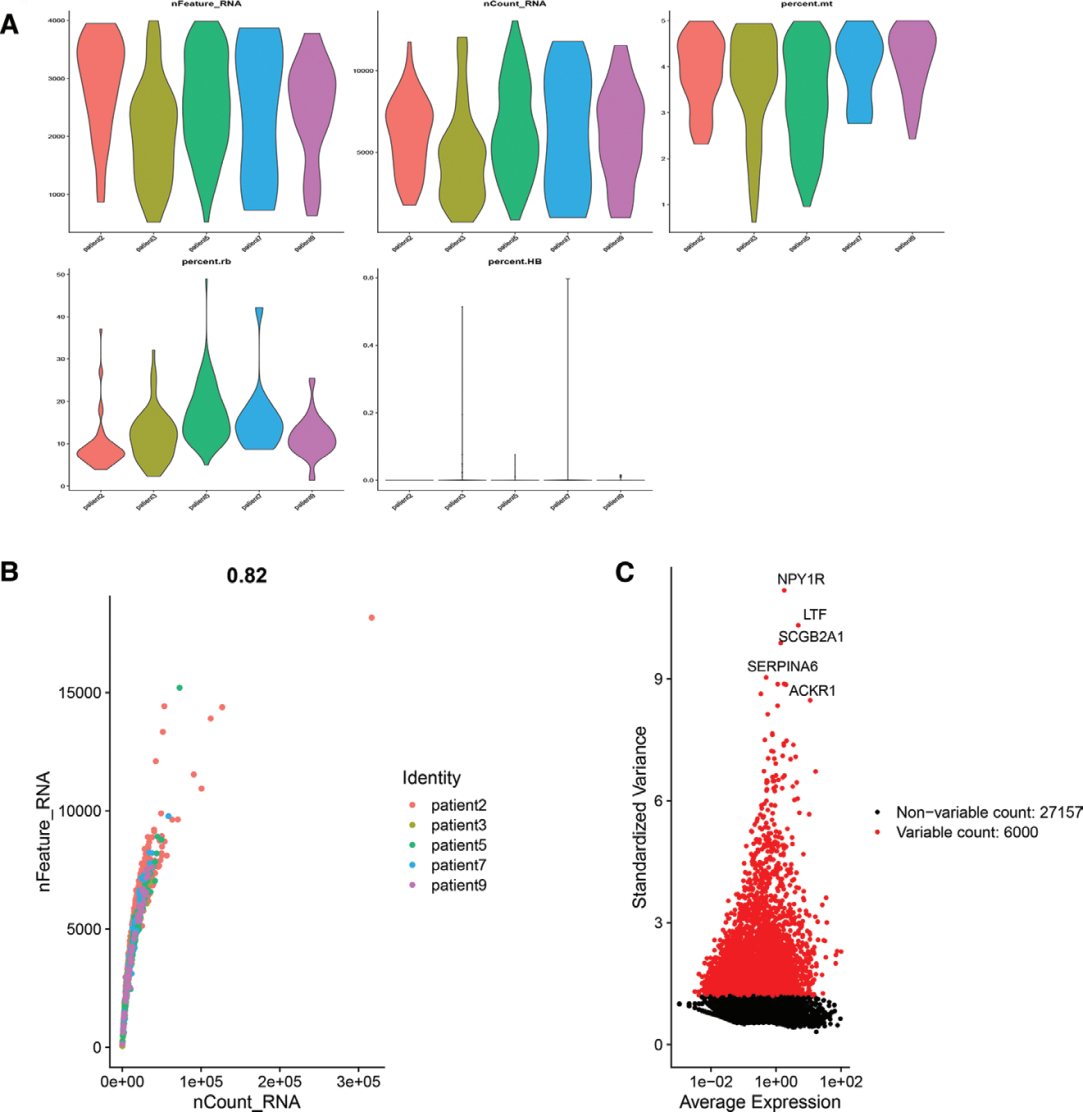
Quality Control. (A) The amount of gene expression per cell. Each cell’s gene expression ranged between 500 and 4000 in the 5 samples. The percentages of erythrocyte and ribosomal genes were < 1%, and mitochondrial genes were < 5%. (B) The cells were distributed evenly among the 5 samples. With a correlation coefficient of 0.82, the number of genes and their expression levels are positively correlated. (C) From all genes, we chose 6000 hypervariable genes, which were highlighted in red. We also marked the top 5 genes.

**Figure 3.**
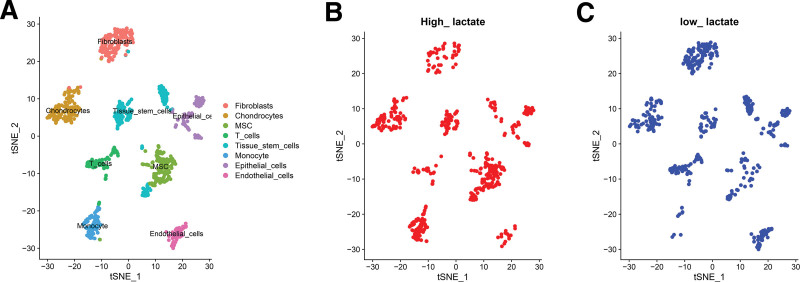
Single-cell sequencing analysis. (A) The cells were classified as Fibroblasts, monocytes, Endothelial_cells, Epithelial_cells, Tissue_stem_cells, and Chondrocytes based on surface marker genes for several cell types. (B, C) Distribution of high_ and low_ lactate cells. High and low lactate cells were relatively evenly distributed across the cell clusters.

Each cell’s gene expression ranged between 500 and 4000 in the 5 samples, which was rather uniformly distributed. Meanwhile, the percentages of erythrocyte and ribosomal genes were < 1%, and mitochondrial genes were < 5% (Fig. 2A). With a 0.82 correlation value, Figure 2B showed there was a positive correlation between gene number and expression. Figure 2C showed of all genes, 6000 hypervariable genes were screened and the top 5 hypervariable genes were marked. After integrating them, we classified all cells into 13 categories using the principal component analysis approach and the TSNE clustering methodology (Fig. 3A). We used the “PercentageFeatureSet” function to input 4 prognostic lactate metabolism genes and got their proportions. We divided them into low lactate or high lactate cells according to the median ratio, which was relatively evenly distributed in each cell cluster (Figs. 3B, 3C). Finally, we performed a differential analysis of gene expression between the 2 groups and obtained a total of 450 LMRG.

### 3.3. Construction and Evaluation of the Model2

**Figure 4.**
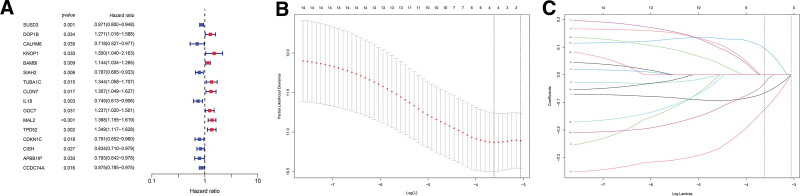
Model2 construction. (A) Univariate COX analysis. 16 prognostic genes in all were selected. Genes in red are high risk, whereas genes in blue are low risk. (B, C) LASSO regression analysis. Further prognostic gene selection was carried out using LASSO regression analysis. The curve converged when Lamda was 4. LASSO = the least absolute selection operator.

We performed differential analysis on the matched data to screen out 114 genes that differed between normal and tumor groups. Through independent prognostic analysis, we screened 16 LMRG (Fig. 4A). Then through the least absolute selection operator regression analysis, we further screened out 4 LMRG and constructed the Model2 (Figs. 4B, 4C). These calculations were made for the Model2: risk score = -SUSD3*0.06587582 - IL18*0.12647563 + MAL2*0.09540324 - CDKN1C*0.03048204.

**Figure 5.**
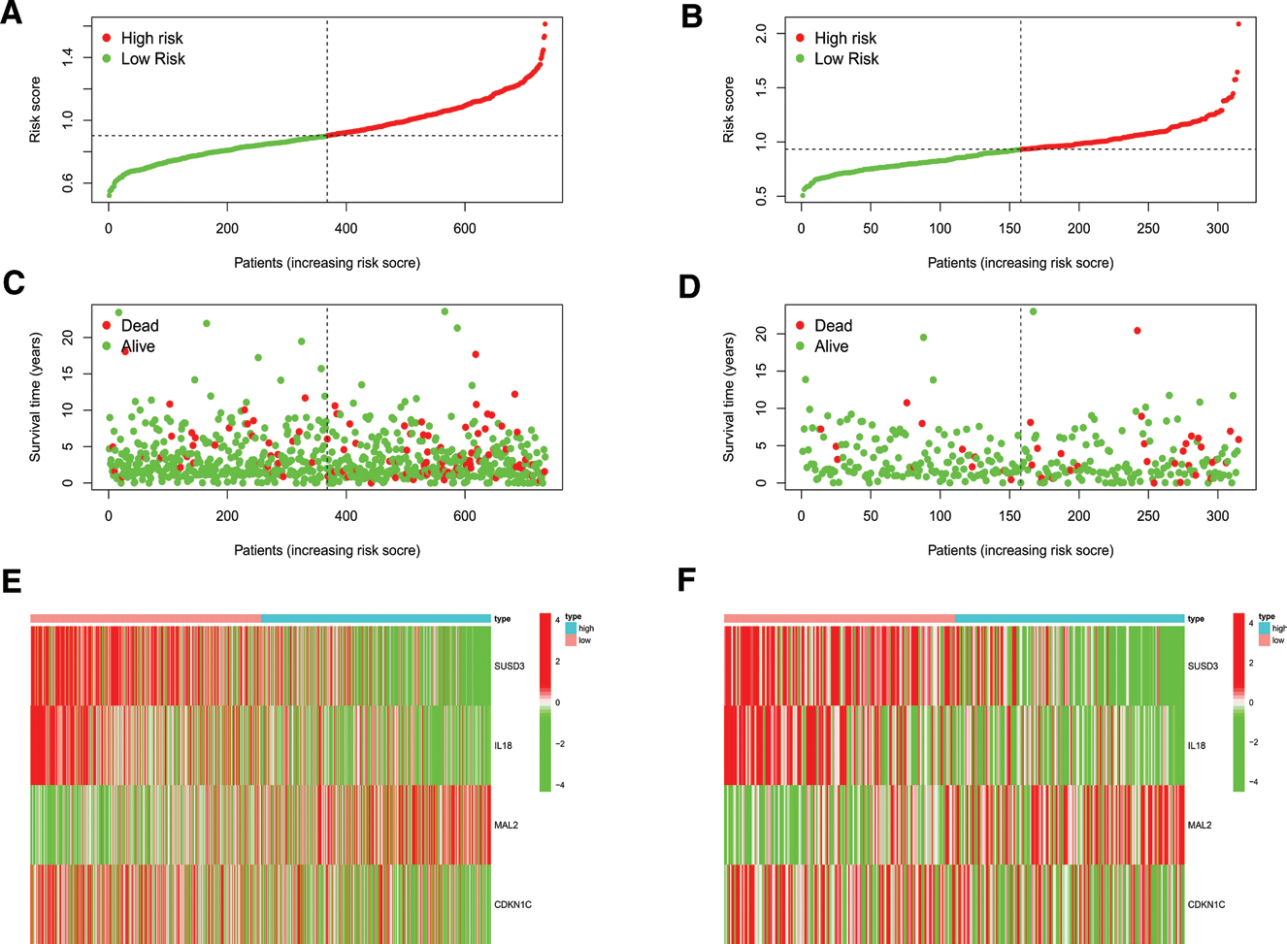
Model2 evaluation. (A, B) The risk score of training (A) and test (B) cohorts. We separated them into high- and low-risk groups using the median. (C, D) The correlation between survival status and risk score. The percentage of patients that died increased with a risk score. (E, F) Expression heatmap of 4 model genes. In the high-risk group, MAL2 was substantially expressed. In low-risk populations, SUSD3, IL18, and CDKN1C were highly expressed.

**Figure 6.**
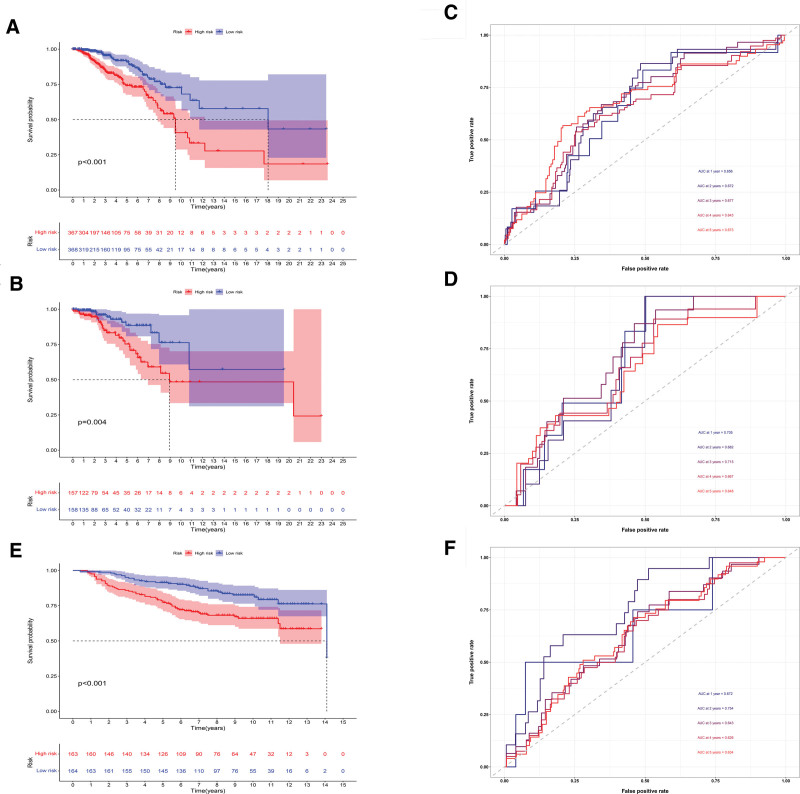
Model2 evaluation. (A, B) In both training (A) and test (B) cohorts, high-risk patients had poor outcomes. (C, D) In both cohorts, the AUC was in the range of 0.6 to 0.7. (E, F) The result for high-risk groups was poorer in the validation cohort. The AUC was in the range of 0.6 to 0.7. AUC = the area under the curve.

Figures 5A and 5B showed that we separated breast cancer patients into high- and low-risk groups. The percentage of patients that died increased with risk score (Figs. 5C, 5D). Besides, among those at high risk, MAL2 was substantially expressed. (Figs. 5E, 5F). Among those at low risk, SUSD3, IL18, and CDKN1C were highly expressed (Figs. 5E, 5F). The outcome was substantially worse for the high-risk group, according to the findings of the survival study (Figs. 6A, 6B). For the training cohort, the area under the curves (AUCs) were 0.656, 0.672, 0.677, 0.643, and 0.673 at 1, 2, 3, 4, and 5 years, respectively (Fig. 6C). For the test cohort, the AUC were 0.705, 0.682, 0.715, 0.667, and 0.648 at 1, 2, 3, 4, and 5 years, respectively (Fig. 6D). Additionally, the model correctly classified the patients in the validation cohort (Fig. 6E). The AUCs were 0.672, 0.754, 0.643, 0.626, and 0.634 at 1, 2, 3, 4, and 5 years, respectively (Fig. 6F).

### 3.4. Functional analysis

**Figure 7.**
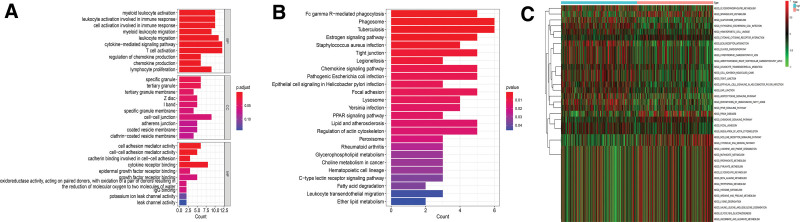
Functional analysis. (A) According to the findings of the GO enrichment analysis, the major functions of these genes were the activation of immune cells, immune response, and cell adhesion. (B) According to the findings of the KEGG enrichment analysis, these genes were mainly involved in immune responses and cell-to-cell connections. (C) These genes were mainly involved in lipid metabolism and cell-to-cell interactions according to the results of the GSVA analysis. GO = the gene ontology, GSVA = gene set variation analysis, KEGG = kyoto encyclopedia of genes and genomes.

According to the findings of the gene ontology enrichment analysis, the major functions of these genes were the activation of immune cells, immune response, and cell adhesion (Fig. 7A). According to the findings of the kyoto encyclopedia of genes and genomes enrichment analysis, these genes were mainly involved in immune responses and cell-to-cell connections, (Fig. 7B). These genes were mainly involved in lipid metabolism and cell-to-cell interactions according to the results of the GSVA analysis (Fig. 7C).

### 3.5. Immunoassay and m6A analysis

**Figure 8.**
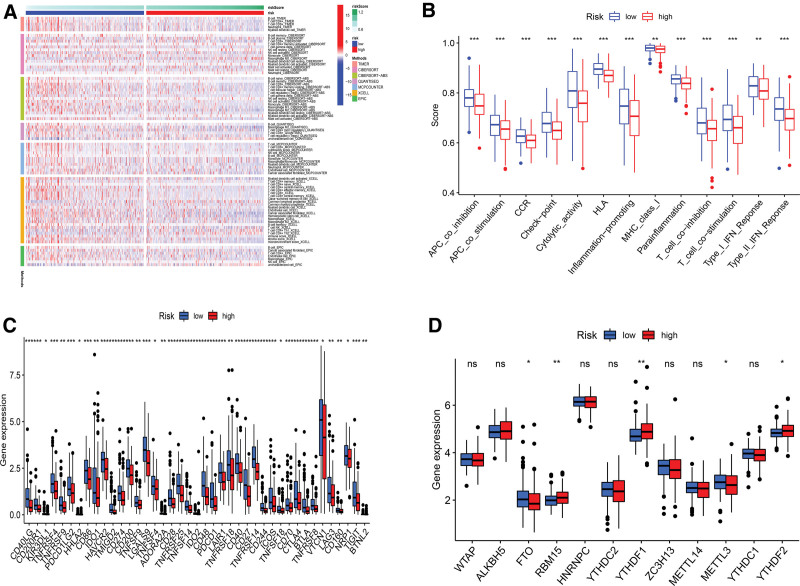
Immunoassay and m6A analysis. (A) NK cell, B cell, T cell, and Myeloid dendritic were significantly related to risk scores. (B) The immune function is more active in low-risk groups. (C) The majority of immunological checkpoint genes were up-regulated. (D) M6A-related gene expression. The expression of FTO, RBM15, YTHDC2, METTL3, and YTHDF2 differed.

Tumor growth depends heavily on the immunological microenvironment. Immunocorrelation analysis showed NK cell, B cell, T cell, and Myeloid dendritic cell were strongly correlated with risk scores (Fig. 8A). Figures 8B and 8C showed that significant variations in immunological checkpoint gene expression and immune response existed between the 2 groups. We explored m6A-related genes to better guide immunotherapy. The results showed that the expression of m6a-related genes varied (Fig. 8D).

### 3.6. Construction of the Model3

**Figure 9.**
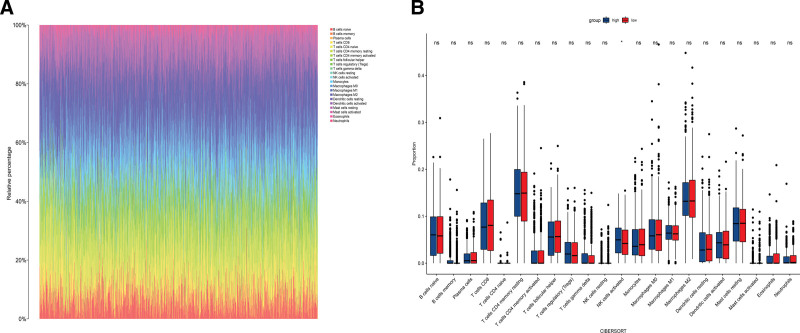
Immune cell infiltration. (A) The percentage of each type of immune cell in each sample. (B) Immune cell differences between the 2 groups. The NK cells activated were significantly different.

22 immune cells were obtained by CIBERSORT analysis. Figure 9A showed the number of various immune cells present in each sample. Patients were split into high-expression and low-expression groups based on the median expression of NDUFAF6. The results showed that the NK cells activated were significantly different (Figure 9B). The NK cells activated were identified as LMIC. We used the value of NK cells activated as Model3.

### 3.7. Combined prognostic model

**Figure 10.**
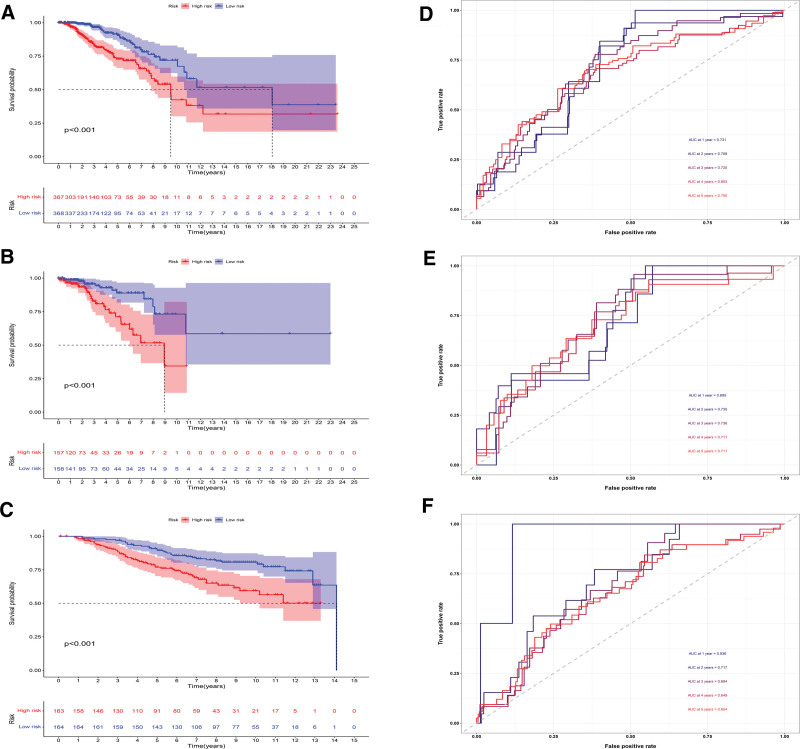
The combined model evaluation. (A-C) High-risk patients had worse prognoses in all three cohorts. (D-F) The AUC was basically between 0.7 and 0.9 in all three cohorts. AUC = the area under the curve.

By multivariate COX regression, we established the combined prognostic model. The combined model was computed using the formula: combined score = Model1*0.2104 to Model2*2.0594 to Model3*2.2000. The high-risk patients fared substantially worse (Figs. 10A–10C). For the training cohort, the AUC were 0.731, 0.708, 0.720, 0.683, and 0.700 at 1, 2, 3, 4, and 5 years, respectively (Fig. 10D). For the test cohort, the AUCs were 0.695, 0.735, 0.736, 0.711, and 0.711 at 1, 2, 3, 4, and 5 years, respectively (Fig. 10E). For the validation cohort, The AUCs were 0.936, 0.717, 0.694, 0.649, and 0.654 at 1, 2, 3, 4, and 5 years, respectively (Fig. 10F). The AUC was essentially in the range of 0.7 to 0.8 in three cohorts, indicating this model was efficient, robust, and outperformed Model2.

### 3.8. Drug sensitivity analysis

**Figure 11.**
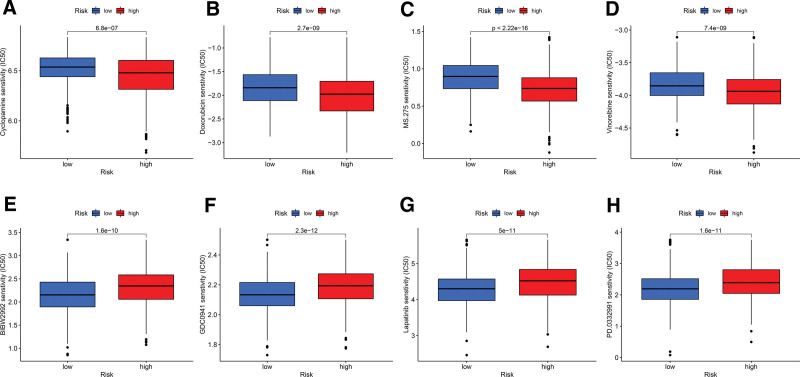
Drug sensitivity analysis. (A-D) The high-risk group was more sensitive to Cyclopamine (A), Doxorubicin (B), Entinostat (C), and Vinorelbine (D). (E-H) The low-risk group was more sensitive to Afatinib (E), Pictilisib (F), Lapatinib (G), and Palbociclib (H).

For targeted therapy, we perform drug susceptibility testing to identify effective drugs. After analysis, in the high-risk group, Cyclopamine, Doxorubicin, Entinostat, and Vinorelbine were more sensitive (Figs. 11A–11D). In the low-risk group, Afatinib, Pictilisib, Lapatinib, and Palbociclib were more sensitive (Figs. 11E–11H).

## 4. Discussion

In this study, we conducted in-depth bioinformatics research to examine the importance of lactate metabolism genes, lactate metabolism-related genes, and lactate metabolism-related immune-infiltrating cells in breast cancer. Based on TCGA and GEO databases, we constructed Model1 based on NDUFAF6 expression, prognostic Model2 based on LMRG, and Model3 based on NK cells activated value. We further build a combined model based on the above models, which can accurately stratify breast cancer patients and predict their prognosis. At the same time, we found that the combined model outperformed the LMRG-based prognostic model in terms of predictive performance. Our study also showed significant differences in the role of LMRG in the immune microenvironment of breast cancer, which might provide breast cancer patients with a new predictor for treatment. Drug sensitivity analysis helped stratify the therapy of breast cancer by identifying more sensitive medications.

The tumor microenvironment is crucial in tumor growth by providing energy and nutrition to support tumor growth and metastasis.^[31]^ Tumor cells use excess nutrients and oxygen to increase the production of harmful metabolic byproducts, particularly lactic acid, in the tumor microenvironment.^[14]^ Hypoxia is a key feature of breast cancer.^[32]^ Many studies have shown that lactate metabolism is altered in breast cancer, making lactate metabolism a biomarker in breast cancer diagnosis, treatment, and prognosis prediction.^[33,34]^ However, there are no studies on lactate metabolism genes in breast cancer. We provide for the first time a prognostic signature of lactate metabolism genes and LMRG in breast cancer, which have important implications for breast cancer prognosis.

The development and course of the illness have been associated with 5 genes in the combined model, according to preliminary findings. NDUFAF6 is a key gene associated with the mitochondrial translation process, which is a potential prognostic and key gene in hepatocellular carcinoma.^[35]^ SUSD3 is a protein located in the plasma membrane.^[36]^ Aushev et al confirmed that SUSD3 is connected to breast cancer prognosis.^[37]^ IL18 is a pro-inflammatory cytokine.^[38]^ IL18 is a crucial component in the 6-gene prognostic model developed by Xiao et al to assess the prognosis of esophageal squamous cell carcinoma.^[39]^ MLA2 is a multi-transmembrane protein.^[40]^ Lu et al confirmed that MAL2 promoted the development of cervical cancer.^[41]^ CDKN1C encodes p57 and promotes bladder cancer progression.^[42]^ In this study, the combined prognostic model includes these 5 genes and can better help us understand tumor progression.

Studies have confirmed that an important marker of tumor progression is tumor immune escape.^[43]^ Evasion of the immune response allows the tumor to grow.^[44]^ Although the outcomes of clinical trials indicate that immunotherapy may be a cutting-edge strategy for treating breast cancer, a considerable number of patients are not sensitive to immunotherapy.^[45,46]^ We think that the high levels of lactate due to hypoxia play an important role in drug resistance in breast cancer. Hypoxia in the breast cancer immune microenvironment affects tumor cells and stromal cells.^[47]^ Tumor cells in a hypoxic environment are highly aggressive and resistant to immunotherapy.^[48]^ The study confirmed that high levels of lactic acid can lead to chemoresistance in breast cancer.^[49]^ Understanding lactate metabolism and the immunological microenvironment in breast cancer is crucial. Based on lactate metabolism genes, we constructed a combined model. Between high- and low-risk groups, we discovered a substantial difference in immune cell infiltration and immune checkpoint gene expression, which could guide immunotherapy in breast cancer patients. Our study also selected drug candidates for each of the 2 groups of patients, which had important implications for guiding the clinical management of breast cancer patients.

M6A is the most common RNA modification and plays important role in various cellular pathways and biological processes.^[50,51]^ Certain cancers and aberrant immunological modulation are both attributed to factors that influence m6A alteration.^[52,53]^ M6A modifications are closely related to the tumor microenvironment.^[23]^ Besides, m6A regulator-mediated tumor microenvironment cell infiltration features are beneficial for tumor immunotherapy.^[54]^ Our study analyzed m6A-related genes, which could help screen patients sensitive to immunotherapy.

Tumor immune infiltrating cells play a critical role in the tumor immunological microenvironment. An increasing number of studies implicate tumor immune-infiltrating cells in response to chemotherapy and immunotherapy.^[55]^ T-cell-based immunotherapy has been used for a variety of cancers, but overall, only a small percentage of patients respond to treatment.^[56,57]^ Unlike T cells which require MHC molecules to deliver tumor antigens, NK cells are lymphocytes of the innate immune system, capable of spontaneously detecting and killing tumor cells.^[58]^ Besides, preclinical evidence proves NK cell-based immunotherapy is a safe and feasible therapeutic strategy.^[59]^ At the same time, the study of Goff et al confirmed that the development and spread of breast cancer are significantly influenced by NK cells.^[60]^ Therefore, NK cell-based immunotherapy may be a promising therapeutic approach for breast cancer. This study can screen breast cancer patients who are sensitive to NK cell-based immunotherapy and help stratify the treatment of breast cancer patients.

To our knowledge, this is the 1^st^ model constructed using lactate metabolism genes, LMRG, and LMIC. It gives data for the research of breast cancer tumor metabolism and is useful for breast cancer sufferers’ therapy.

## 5. Conclusion

Based on lactate metabolism genes, lactate metabolism-related genes, and immune infiltrating cells, a combined prognostic model of breast cancer was established. Using this model, the breast cancer patient’s prognosis and immunological microenvironment can be correctly estimated. Besides, our discoveries might lead to new methods of breast cancer treatment.

## Acknowledgments

We are very grateful for the data provided by databases such as TCGA, and GEO.

## Author contributions

**Funding acquisition:** Jianping Zhang.

**Methodology:** Na Lu, Xiao Guan, Wei Bao, Zongyao Fan.

**Validation:** Jianping Zhang.

**Writing – original draft:** Na Lu, Xiao Guan, Wei Bao, Zongyao Fan.

**Writing – review & editing:** Jianping Zhang.
